# Unusual presentation of benzodiazepine withdrawal with Takotsubo syndrome: a case report

**DOI:** 10.1093/ehjcr/ytae136

**Published:** 2024-03-18

**Authors:** Isabel Durães Campos, Helena Moreira, Francisco Portal, José Artur Paiva

**Affiliations:** Department of Intensive Care Medicine, Centro Hospitalar Universitário São João, Alameda Prof. Hernâni Monteiro, Porto 4200-319, Portugal; Department of Cardiology, Centro Hospitalar Universitário São João, Porto, Portugal; Department of Interne Medicine, Centro Hospitalar Universitário São João, Porto, Portugal; Department of Intensive Care Medicine, Centro Hospitalar Universitário São João, Alameda Prof. Hernâni Monteiro, Porto 4200-319, Portugal

**Keywords:** Takotsubo syndrome, Benzodiazepine withdrawal, Benzodiazepine withdrawal–induced Takotsubo syndrome, Altered mental status, Case report

## Abstract

**Background:**

Thousands of people suffer from anxiety, depression, and insomnia every day, with benzodiazepines being one of the strategies used to treat these conditions. Withdrawal from its long-term use can lead to potentially life-threatening complications, including Takotsubo syndrome. The authors highlight an atypical case of Takotsubo syndrome secondary to benzodiazepine withdrawal, a rare life-threatening complication of acute substance withdrawal.

**Case summary:**

A 58-year-old female presented to the emergency department with altered mental status and acute pulmonary oedema after discontinuing her prescribed benzodiazepines 3 days prior to presentation. Electrocardiogram (ECG) demonstrated anterior ST-segment elevation, with Q-wave and T-wave inversion with prolonged QT interval. Troponin I concentration and B-type natriuretic peptide were elevated to 5407 ng/L (normal ≤ 16 ng/L) and to 1627.0 pg/L (normal ≤ 100 pg/mL), respectively. Echocardiogram showed ballooning of the left ventricle (LV) apex with dyskinesia of the mid and apical segments, with LV function of 15%. Coronary angiography was normal, but left ventriculography showed severe LV systolic dysfunction with akinesis of the mid and apical LV segments and hyperdynamic basal segments. A presumptive diagnosis of benzodiazepine withdrawal–induced Takotsubo syndrome was made, and patients’ symptoms, ECG findings, and LV dysfunction resolved after benzodiazepine administration. Six months post discharge, the patient remained asymptomatic with a normal biventricular function, and a beta-blocker was successfully introduced as part of a lifelong plan.

**Discussion:**

A diagnosis of benzodiazepine withdrawal–induced Takotsubo syndrome is an underrecognized and challenging diagnosis, due to its atypical clinical presentation. High degree of clinical suspicion for this syndrome is crucial, since favourable prognosis depends on prompt diagnosis and treatment.

**Learning points**

- Benzodiazepine withdrawal–induced Takotsubo syndrome is a possible life-threatening complication of acute benzodiazepine withdrawal rarely described in the literature.
- As the clinical presentation can be atypical, it is crucial that clinicians maintain a high degree of clinical suspicion for this syndrome.
- This diagnosis should be suspected in patients with acute benzodiazepine withdrawal who present with altered mental status, tachycardia, changes in blood pressure, and/or dyspnoea.
- The gold standard diagnostic tool is the disproportion between cardiac troponin level and B-type natriuretic peptide level, electrocardiogram, echocardiogram, and coronary angiography.
- Appropriate withdrawal management is a vital component of care for withdrawal-associated Takotsubo syndrome.
- β-blockers are the most used cardiac medications, given the probable mechanism of increased catecholamine action.

## Introduction

Thousands of people suffer from anxiety, depression, and insomnia, with benzodiazepines being one of the strategies used to treat these conditions.^1^ Since 2010 across Europe, there has been an increase in the use of psychoactive drugs, namely benzodiazepine prescriptions.^2^ This was observed even more since the beginning of COVID-19 pandemic.^3^ These sedatives may not seem dangerous at first; however, withdrawal from long-term use can lead to potentially life-threatening complications, including Takotsubo syndrome.^4^ As the clinical presentation of benzodiazepine withdrawal–induced Takotsubo syndrome can be atypical, it is important to have a high degree of clinical suspicion for this diagnosis in patients with suspected acute benzodiazepine withdrawal, who present with altered mental status, tachycardia, changes in blood pressure, and/or dyspnoea.

## Summary figure

**Table:** 

Prior to admission	The patient discontinued alprazolam 3 days prior to admission.
Day 0 (at home)	The patient presented altered mental status after generalized tonic–clonic seizure and received 10 mg of IV diazepam and 500 mg of levetiracetam.
Day 0 (emergency department)	Acute pulmonary oedema with a slightly elevated troponin I concentration (5407 ng/L) and B-type natriuretic peptide (1627.0 µg/L).
	Transthoracic echocardiogram revealed a ballooning of the left ventricle apex with dyskinesia of the mid and apical segments (ejection fraction 15%). Coronary angiography showed no abnormalities. Left ventriculography showed severe left ventricular (LV) systolic dysfunction with akinesia of the mid and apical LV segments and hyperdynamic basal segments.
	A presumptive diagnosis of Takotsubo syndrome secondary to benzodiazepine withdrawal was made.
Day 0 (intensive care unit)	Administration of diazepam 10 mg intravenous (three times daily).
Day 7	Hospital discharge, with alprazolam (2 mg/day). Follow-up transthoracic echocardiogram showed improvement of LV function with complete resolution of the wall motion abnormalities.
6-month follow-up	Asymptomatic with a normal biventricular function. Beta-blocker was successfully introduced as part of a lifelong plan.

This case report presents an uncommon scenario of Takotsubo syndrome due to benzodiazepine withdrawal, which presented with altered mental status associated with acute pulmonary oedema and electrocardiogram changes mimicking acute coronary syndrome.

## Case presentation

A 58-year-old female with major depressive disorder, bronchiectasis, and osteoporosis and without a relevant cardiovascular history was admitted to the emergency department (ED) with altered mental status after generalized tonic–clonic seizure at home. History obtained from the patient’s family revealed that the patient had been taking alprazolam for over 10 years, but she had discontinued the prescribed dose of this drug (3 mg two twice daily) 3 days prior to admission, after which her condition began to deteriorate. Additional history provided by family members indicated that the patient was taking additional alprazolam, acquired without a prescription (20–30 mg/day).

**Figure 1:**
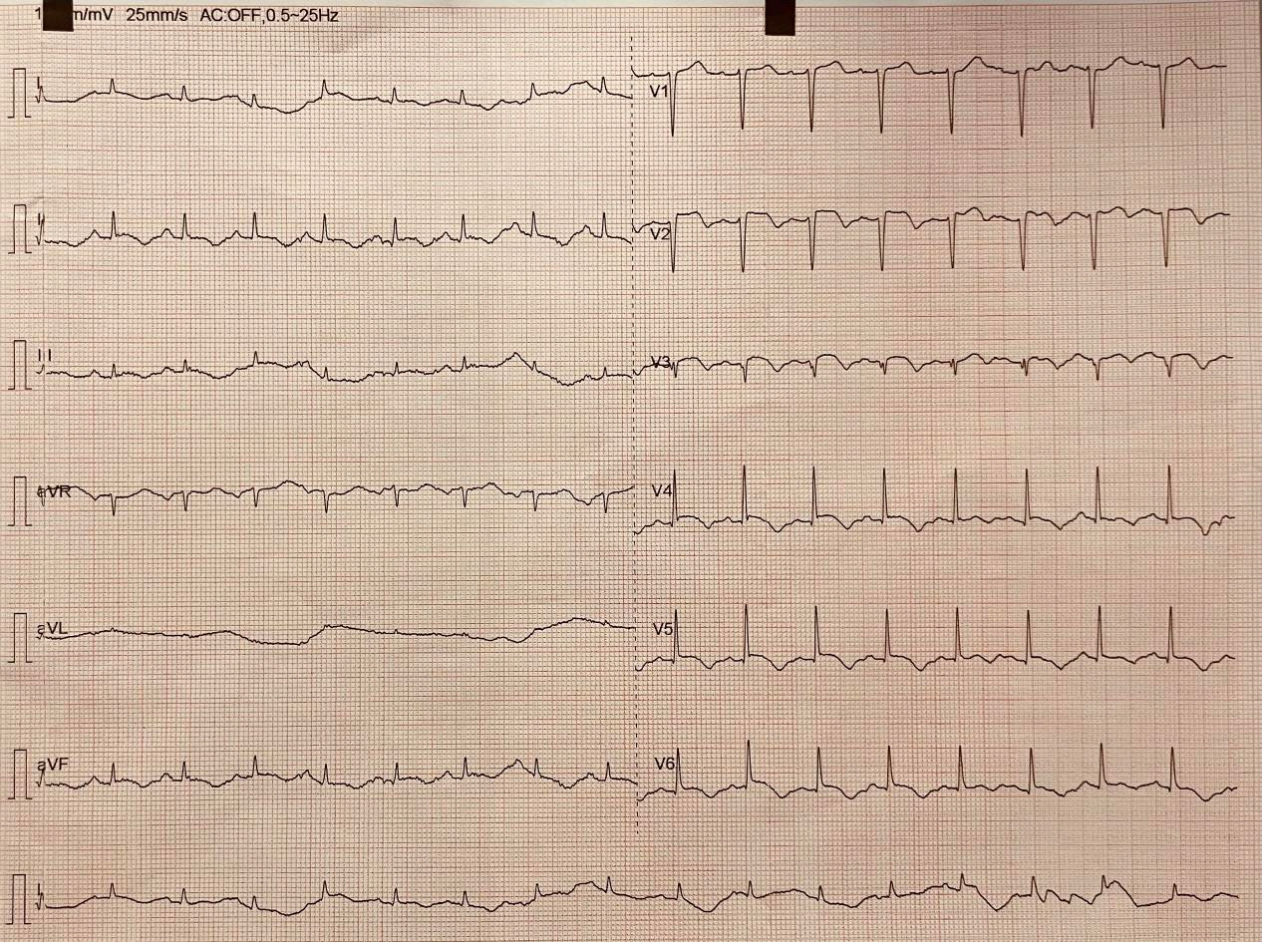
Electrocardiogram showing sinus tachycardia, anterior ST-segment elevation with Q-waves, and T-wave inversion with prolonged QT interval.

On initial examination at home, the patient was found to be tremulous and somnolent, having received 10 mg of IV diazepam and 500 mg of levetiracetam. Physical examination on admission at the ED revealed a decreased level of consciousness, a pulse rate of 127 b.p.m., temperature of 37.4°C, blood pressure of 153/87 mmHg, and respiratory rate of 27 breaths per minute with decreased oxygen saturation to 87% on room air. The remaining physical examination and thoracic radiographic examination revealed the presence of acute pulmonary oedema (rales and pulmonary interstitial oedema, respectively). Following initial treatment, including non-invasive pressure support ventilation and administration of intravenous boluses of furosemide (60 mg) and isosorbide dinitrate (2 mg), the patient showed rapid improvement with led dependency on oxygen supplementation therapy and resolution of tachycardia and arterial hypertension. Blood analyses performed in the ED were overall unremarkable, except for a slightly elevated troponin I concentration (5407 ng/L; normal ≤ 16 ng/L), creatinine kinase (CK) total (5607 U/L; normal: 10–149 U/L), and B-type natriuretic peptide (BNP) (1627 pg/L; normal ≤ 100 pg/mL). Additional laboratory analyses showed elevated acute phase reactants (mild leucocytosis and C-reactive protein of 7.7 mg/L). Investigation for cerebrovascular accident and infectious aetiologies was also unremarkable. Urine analyses with toxicology screen were positive for benzodiazepines. The electrocardiogram (ECG) (*Figure 1*) showed sinus tachycardia, anterior ST-segment elevation with Q-waves, and T-wave inversion with prolonged QT interval. Transthoracic echocardiogram (TTE) revealed ballooning of the left ventricle (LV) apex with dyskinesia of the mid and apical segments (see Supplementary material online, *Video S1*). The overall LV ejection fraction was estimated of 15%. Given the suspicion of a ST-elevation myocardial infarction, an emergency coronary angiography was performed showing no abnormalities, and left ventriculography showed severe LV systolic dysfunction with akinesia of the mid and apical LV segments and hyperdynamic basal segments (see Supplementary material online, *Videos S2* and *S3*). A presumptive diagnosis of Takotsubo syndrome secondary to benzodiazepine withdrawal was made. Moreover, a gradual return to baseline was noted with administration of diazepam 10 mg intravenously three times daily. No heart failure prognosis-modifying drugs were prescribed, given the patient’s low blood pressure profile (systolic blood pressure < 90 mmHg) and her favourable evolution. Medication at discharge was only alprazolam (2 mg/day).

At discharge, after 7 days of hospitalization, the ECG showed normalization of the QT interval, the TTE showed improvement of LV function with complete resolution of the wall motion abnormalities (see Supplementary material online, *Video S4*), and the diagnosis of Takotsubo syndrome secondary to benzodiazepine withdrawal was confirmed. After 6 months, the patient remained asymptomatic with a normal biventricular function, and a beta-blocker was successfully introduced as part of a lifelong plan.

## Discussion

The authors highlight an atypical case of Takotsubo syndrome secondary to benzodiazepine withdrawal, a possible life-threatening complication of acute substance withdrawal rarely described in the literature.^4,5,6,7^

Takotsubo syndrome is an uncommon heart failure syndrome characterized by acute transient systolic dysfunction with a peculiar LV wall motion abnormality, with a prevalence of 1.0–2.5%.^8^ Although its presentation is difficult to distinguish from acute coronary syndrome (similar symptoms at presentation, ECG abnormalities, and elevated cardiac biomarkers), Takotsubo syndrome differs from the latter by the absence of obstructive coronary artery disease or plaque rupture. It usually appears after a significant physical and emotional stressor; however, reports of acute benzodiazepine withdrawal–induced Takotsubo syndrome are emerging.^4^ This acute heart failure syndrome is increasingly recognized after acute neurological disorders, being identified in 6.7% as the preceding triggering factor. The most common neurological disorders are seizures, intracranial haemorrhage, and ischaemic stroke.^9,10,11^ The pathophysiology of Takotsubo syndrome is not completely understood, but it appears that an excess of catecholamines is implicated in the setting of benzodiazepine withdrawal, resulting in cardiac stunning, which then creates transient systolic and diastolic LV dysfunction with wall motion abnormalities.^5^ Prolonged elevation of circulating catecholamines for several hours has also been reported after other acute neurological disorders such as seizures and stroke.^12,13^

In a review of withdrawal-associated Takotsubo cases, patients mostly presented with withdrawal symptoms such as tachycardia (65.2%), changes in blood pressure (47.8%), and altered mental status (47.8%), with dyspnoea (34.8%), chest pain (26.1%), and nausea (26.1%) presenting less commonly.^4^ These atypical presentations may result from the patients’ inability to accurately report their symptoms in the setting of acute withdrawal, given that many of them present with an altered state of consciousness, as in this case report. This diagnosis is also important in patients that experience seizures, another known trigger for the development of Takotsubo syndrome.^14^ Myocardial infarction with non-obstructive coronary arteries (MINOCA) was a differential diagnosis considered in this clinical case. However, in this pathology, the changes in electrocardiographic and echocardiographic examinations do not resolve after a week.^15^ In this clinical case, if there was no complete resolution from the electrocardiographic and echocardiographic findings, it would be worth considering cardiac magnetic resonance imaging to better clarify the cardiac dysfunction.

Despite strong similarities between Takotsubo syndrome and acute coronary syndrome, there is no universally recommended treatment for this disease. However, in benzodiazepine withdrawal–induced Takotsubo syndrome, it is believed that adequate withdrawal management is crucial in order to not to prolong the state of catecholamine excess, which may result in further catecholamine-mediated cardiotoxicity.^16^ In addition to providing benzodiazepine withdrawal treatment, β-blockers are reportedly the most used cardiac medications in these patients, given the probable mechanism of increased catecholamine action.^4^

Therefore, sharing this underrecognized and rarely described diagnosis is relevant for all physicians who work in the emergency department, as it alerts professionals to this pathology and its early signs (e.g. altered mental status, tachycardia, changes in blood pressure, and/or dyspnoea). It is essential to share more case reports with withdrawal-associated Takotsubo syndrome and the different presentations that these patients may have, in order to increase the sensitivity of their diagnosis.

## Conclusion

Benzodiazepine withdrawal–induced Takotsubo syndrome is a rare and potentially fatal complication, difficult to diagnose due to the atypical clinical presentation. The authors describe a case report consistent with Takotsubo syndrome during acute benzodiazepine withdrawal, with the patients’ symptoms, ECG findings, and LV dysfunction that mostly resolved after benzodiazepine administration. Thus, a high degree of clinical suspicion for withdrawal-associated Takotsubo syndrome in patients with a history of substance use disorders or physical dependence on benzodiazepines is critical, since favourable prognosis depends on prompt diagnosis and treatment.

## Supplementary Material

ytae136_Supplementary_Data

## Data Availability

The data underlying this article are available in the article and in its online supplementary material.

## Lead author biography

Isabel Durães Campos started her medical training at Institute of Biomedical Sciences Abel Salazar in Porto (Portugal) in 2008. After 1 year of working at Hospital de Braga, she started her cardiology residency in 2016 and finished in 2020. In 2016, she started her PhD in the field of neurocardiology, having already published and presented numerous abstracts at national and international conferences. After 2020, she began her fellowship in critical care medicine at Centro Hospitalar Universitário São João, Porto, Portugal.

## Supplementary material

**Consent:** The authors confirm that written consent for submission and publication of this case report including image(s) and associated text has been obtained from the patient.

**Funding:** None declared.
